# Determinants of professional identity among practitioners in China’s hot spring-integrated care model

**DOI:** 10.3389/fmed.2025.1650338

**Published:** 2025-12-10

**Authors:** Xiaochen Li, Yao Zhou, Jimin Chen, Hongmei Zhang, Qian Liu, Zihan Chen, Xue Yang, Liqing Yao

**Affiliations:** 1 Department of Rehabilitation Medicine, The Second Affiliated Hospital of Kunming Medical University, Kunming, China; 2 School of Rehabilitation Medicine, Kunming Medical University, Kunming, China

**Keywords:** hot spring-integrated care, professional identity, continuing professional development, workforce sustainability, health services research

## Abstract

**Objective:**

To identify key determinants of professional identity among practitioners within China’s Hot Spring Medical and Long-term Care Integration with Rehabilitation Services (HS-MLR) model—an emerging field facing distinct occupational challenges. Understanding these factors is critical for formulating targeted strategies to enhance workforce sustainability.

**Methods:**

A cross-sectional survey was conducted among 221 practitioners across 26 provinces. Data included sociodemographic and occupational characteristics, and professional identity was assessed using a validated multidimensional scale. Exploratory factor analysis (EFA) identified dimensions of identity. Univariate and multivariable stepwise ordinal logistic regression were used to identify independent predictors.

**Results:**

The cohort was predominantly female (57.0%) and relatively young (90.1% aged 26–45 years), with only 16.4% holding a bachelor’s degree or above. EFA revealed two dimensions of professional identity: Professional Value Recognition and Professional Practice Commitment. Multivariable analysis showed that continuing education as an independent predictor of Value Recognition, while perceived treatment effectiveness, social impact, and model acceptance predicted Practice Commitment. Overall, only 47.1% of respondents reported a satisfactory or higher level of professional identity.

**Conclusion:**

Professional identity among HS-MLR practitioners remains moderately low. Key determinants include continuing education, perceived therapeutic efficacy, social value, and belief in the model. A dual-pathway interventions strategy—enhancing continuous professional development and reinforcing perceived social and clinical value—is recommended to improve workforce sustainability and service quality in this emerging sector.

## 1 Introduction

Since the late 20th century, rapid socioeconomic development and rising living standards in China have promoted growing public health demands and a shift from treatment-centered to health-centered models (1). These shifts are not unique to China but mirror a global trend, compelling healthcare systems worldwide to move toward integrated, person-centered models as part of a broader reform of primary health care (PHC). The core objective of PHC reform is to effectively respond to emerging global health challenges such as population aging, multimorbidity, and the increasing prevalence of chronic diseases. A key strategy to achieve this is through system integration, which necessitates moving beyond a traditional focus on physicians and nurses to fully incorporate the expertise of a wider range of health professionals.

In the European context, particularly in Italy, recent studies have extensively analyzed the crucial role that technical, rehabilitation, and preventive health professions (TRP-HPs) play in supporting PHC reform. These works argue that TRP-HPs are essential but often underutilized resources for transitioning from a hospital-centric to a community-based care model (2). These professionals are uniquely positioned to advance reforms through task-shifting, personalizing care pathways, and strengthening community services (3). Furthermore, practical case studies, such as the AgICOT project, have demonstrated that integrating economic and professional resources through multidisciplinary teams is an effective model for ensuring continuity of care for frail populations (4). This international context provides a valuable comparative framework for understanding the emergence of China’s Hot Spring Medical and Long-term Care Integration with Rehabilitation Services (HS-MLR) model, which was developed in response to similar challenges (5, 6).

A distinctive feature of the HS-MLR model is its incorporation of balneotherapy-a non-pharmacological intervention that has gained growing international recognition (7). This is evidenced by long-standing traditions in other countries, such as Japan’s onsen therapy, the state-supported Kur system in Germany and Austria, and France’s thermal treatments, which are often integrated into national healthcare and covered by social insurance (8, 9). Unlike conventional integrated care systems, the clinical effectiveness of this model derives primarily from the unique mechanisms of balneotherapy itself. This approach utilizes the combined physicochemical properties of mineral-rich waters to produce broad therapeutic effects (10), supporting the management of chronic diseases (11, 12), sub-health conditions (13), and psychological disorders (14).

Balneotherapy endows the HS-MLR model with a unique therapeutic breadth, effectively addressing a range of conditions from physical ailments to psychological distress. Physically, the thermal and mechanical effects of hot springs are well-documented to alleviate chronic fatigue and inflammation by modulating metabolic and cellular stress responses (15–17). Chemically, the diverse mineral profiles of different springs offer targeted benefits for cardiovascular, dermatological, and other conditions (18–22). Psychologically, balneotherapy has demonstrated significant positive effects on mental wellbeing. Meta-analysis evidence confirms its efficacy in reducing anxiety and depression and improving sleep quality (14, 23, 24). This holistic, psychophysiological impact is particularly suited to managing the complex interplay between chronic disease, sub-health states, and psychological symptoms (25–30)-setting the HS-MLR model apart from conventional care systems that often treat these issues in isolation.

This distinctive integration of clinical rehabilitation with wellness and hospitality services, however, creates a novel and complex practice environment. Consequently, practitioners may experience role ambiguity and face the challenge of forging a new interdisciplinary professional identity. These dynamics make the study of professional identity not only distinctive but essential to the sector’s sustainable development.

Professional identity-defined as one’s perception and internalization of the values, roles, and norms of their professional group (31)-is a well-established predictor of job satisfaction, performance, and burnout risk in healthcare (32–37). Theoretical framework such as Professional Identity Formation (PIF) posit that identity evolves through dynamic interactions among personal values, professional experiences, and participation in a community of practice (38).

**Figure 1:**
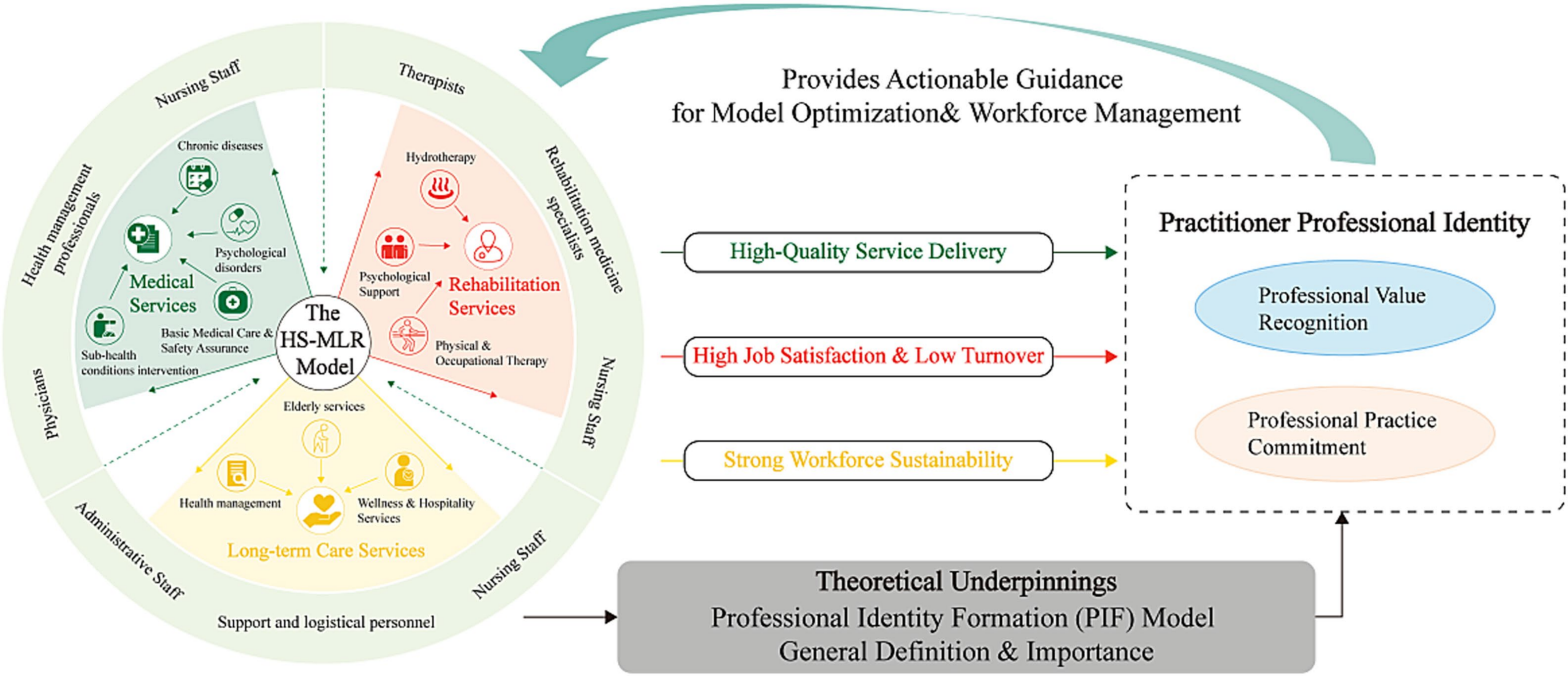
The conceptual framework illustrates the cyclical relationship between the HS-MLR model and practitioners’ professional identity. The Hot Spring Medical and Long-term Care Integration with Rehabilitation Services (HS-MLR) model-characterized by its integrated structure of medical, rehabilitative, and long-term care services and a multidisciplinary workforce-creates inherent demands for high-quality service delivery, high job satisfaction, low turnover, and workforce sustainability. The outcomes are theorized to be mediated by practitioner’s professional identity, a core construct grounded in theories such as Professional Identity Formation (PIF) model. This identity comprises two dimensions identified in this study: Professional Value Recognition and Professional Practice Commitment. A nuanced understanding of this dual-dimensional construct provides actionable guidance that inform the refinement of the HS-MLR model and its workforce strategies, thereby completing a feedback loop that enhance model implementation and stability.

Yet, general models may not fully capture identity constructs within hybrid care settings like HS-MLR. The convergence of clinical, long-term care, and hospitality roles suggests that professional identity in this context may differ markedly from that in conventional healthcare. To explore this empirically, we developed a conceptual framework (Figure 1) guided by the PIF model and Social Exchange Theory. This framework proposes that sociodemographic, occupational, and perceptual factors shape a multifaceted professional identity, which can be elucidated through Exploratory Factor Analysis (EFA). Ultimately, this study aims to inform policy and practice by identifying the specific training and professional development needs of HS-MLR practitioners and exploring the implications for service reorganization required to support this emerging integrated care model.

## 2 Materials and methods

### 2.1 Research subjects

This cross-sectional study was conducted in collaboration with the China Hot Spring Association and focused on institutions implementing the HS-MLR model. A total of 240 standardized questionnaires, entitled *Survey on Practitioners in Chinese Hot Spring Medical and Long-term Care Institutions*, were distributed nationwide. Of these, 221 valid responses were collected, yielding an effective response rate of 92.08%. Questionnaires with more than five missing items or exhibiting patterned or non-serious response were considered invalid and excluded from analysis.

Inclusion criteria were defined at both institutional and individual levels:

(1) Institutional criteria:

- Valid license for hot spring development and utilization, in addition to health and safety certification;
- Continuous operation of hot spring-based wellness programs for at least three years;
- Employment of full-time health management or rehabilitation medicine specialists.

(2) Respondent criteria:

- Sufficient familiarity with institutional operations to complete the questionnaire independently or collaboratively;
- Current employment at the participating institution.

The target sample size was determined according to guidelines for exploratory factor analysis (EFA), which recommends a minimum subject-to-item ratio of 10:1. As the self-developed professional identity scale comprises 7 items, a minimum of 70 participants was required. The final sample of 221 respondents was therefore deemed fully adequate for robust factor analysis.

### 2.2 Ethical declaration

This study was approved by the Ethics Committee of the Second Affiliated Hospital of Kunming Medical University (Approval No. FEY-BG-39-2.0). All participants provided written informed consent and voluntarily participated in the study.

### 2.3 Research methodology and quality control

In July 2024, with support from the Second Affiliated Hospital of Kunming Medical University, Wenzhou Medical University, and the China Hot Spring Association, a project team titled the ‘Hot Spring Medical and Long-term Care Research Group’ was established to investigate this topic.

**Figure 2:**
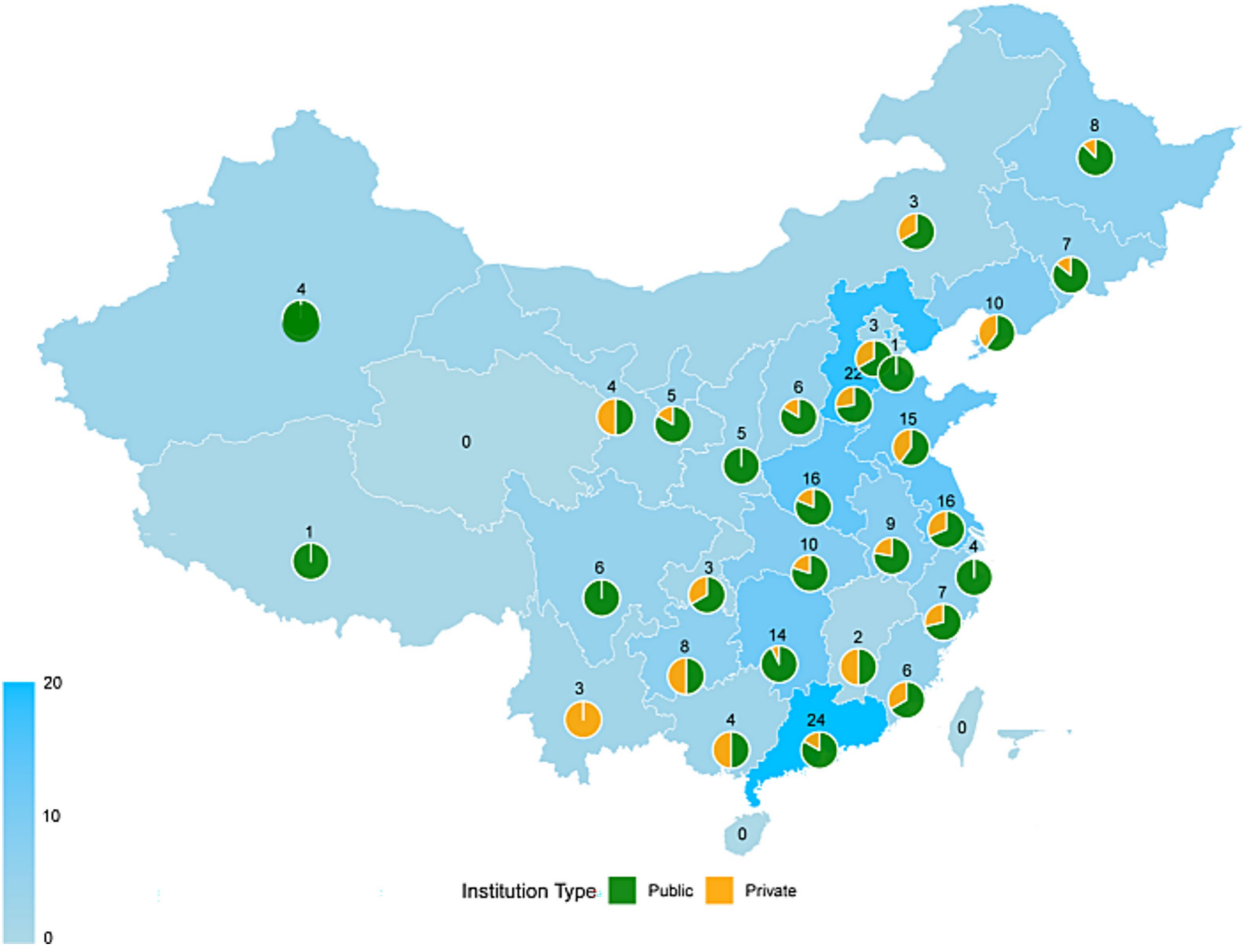
Geographic distribution and ownership type of surveyed institutions across Mainland China. This map displays the provincial distribution of the 221 participating institutions. Regional variation in participation is indicated by color intensity, corresponding to the total number of institutions per province, as shown in the legend. Overlaid pie charts represent the proportion of public (green) and private (orange) institutions within each province. The total number of institutions surveyed per province is indicated adjacent to each pie chart.

To ensure methodological rigor, several measures were implemented. Before commencing the study, all team members underwent standardized training to ensure uniform communication of the research objectives and questionnaire instructions, thereby promoting participants trust and cooperation. To minimize information bias, all surveys were administered independently and anonymously; no personally identifiable information was collected, safeguarding privacy and encouraging candid responses. During data entry, two trained researchers independently entered data into EpiData 3.1 and performed consistency checks. Quality control personnel further reviewed and verified the records to ensure accuracy and reliability. Additionally, to mitigate potential selection bias associated with convenience sampling, we recruited participants from a broad geographic scope covering 26 provinces, encompassing diverse institutional and regional settings (Figure 2). The potential influence of these biases is further discussed in the limitations section.

### 2.4 Instrument development and validation

**Figure 3:**
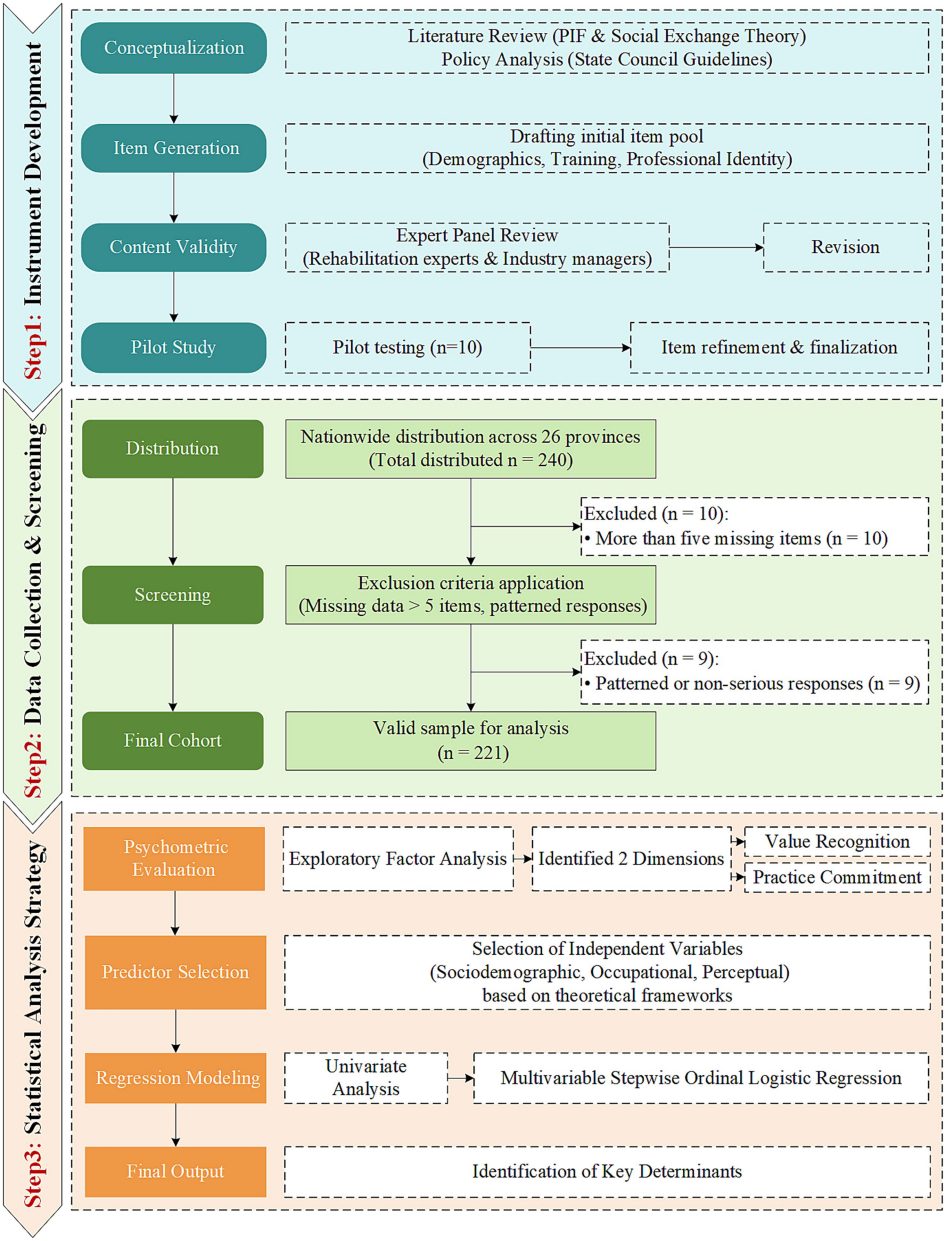
Methodological flowchart of the study. The diagram illustrates the study’s three-stage workflow. Step 1 outlines the instrument development process, from theoretical conceptualization and item generation to expert validation and pilot testing (*n* = 10). Step 2 details the data collection across 26 provinces (*n* = 240) and subsequent screening, which yielded a final valid sample of 221 participants. Step 3 specifies the statistical analysis strategy, beginning with Exploratory Factor Analysis (EFA) to identify the two dimensions of professional identity, followed by univariate and multivariable regression modeling to identify its key determinants.

The survey instrument, entitled *Survey on Practitioners in Chinese Hot Spring Medical and Long-term Care Institutions*, was developed through a rigorous, three-stage process comprising conceptualization, expert validation, and psychometric testing (Figure 3).

Stage 1: Conceptualization and Item Generation. The initial item pool was generated from a comprehensive literature review and policy analysis. We grounded the instrument in the Professional Identity Formation (PIF) framework (32) and Social Exchange Theory to capture multidimensional constructs, including professional values, role perception, and social interaction. Additionally, policy guidelines from the *State Council’s Opinions on Promoting the Integration of Medical Services and Elderly Care* (39) were analyzed to ensure the instrument’s relevance to the specific policy context of China’s HS-MLR model.

Stage 2: Expert Validation. To ensure content validity, a Delphi-like consultation was conducted with an expert panel comprising five specialists: two senior rehabilitation physicians, a professor of statistics, a professor of health management, and a senior executive from the hot spring industry. The panel evaluated each item for relevance, clarity, and conciseness. Based on their feedback, items with ambiguous wording were revised, and redundant items were eliminated. Subsequently, a pilot study was conducted with 10 practitioners from two representative institutions to further refine the instrument’s readability and face validity.

Stage 3: Psychometric Testing. The finalized questionnaire encompassed three domains: demographic characteristics, occupational background, and the professional identity scale. As reported in the Results, the scale demonstrated high internal consistency (Cronbach’s alpha = 0.887) and robust construct validity (KMO = 0.767, *p* < 0.001). Exploratory Factor Analysis (EFA) confirmed a two-factor structure—Professional Value Recognition and Professional Practice Commitment.

The complete questionnaire is provided in Supplementary material 1: Survey Questionnaire.

### 2.5 Pilot study

Prior to the main survey, a pilot study was conducted to capture diversity in geographic location, institutional scale, and service type. Two representative institutions operating under the Chinese Hot Spring Medical and Long-term Care Integration with Rehabilitation Services Model were selected from Yunnan Province—a region known for its abundant hot spring resources—specifically, the Yunnan Provincial Health and Rehabilitation Center and Tengchong Donghu Hot Spring Rehabilitation Medical Center. Ten practitioners from these sites were recruited. Feedback from the pilot phase informed revisions to the questionnaire, including item refinement, elimination, and structure adjustments to improve clarity and facilitate independent completion.

### 2.6 Statistical analysis

**Figure 4:**
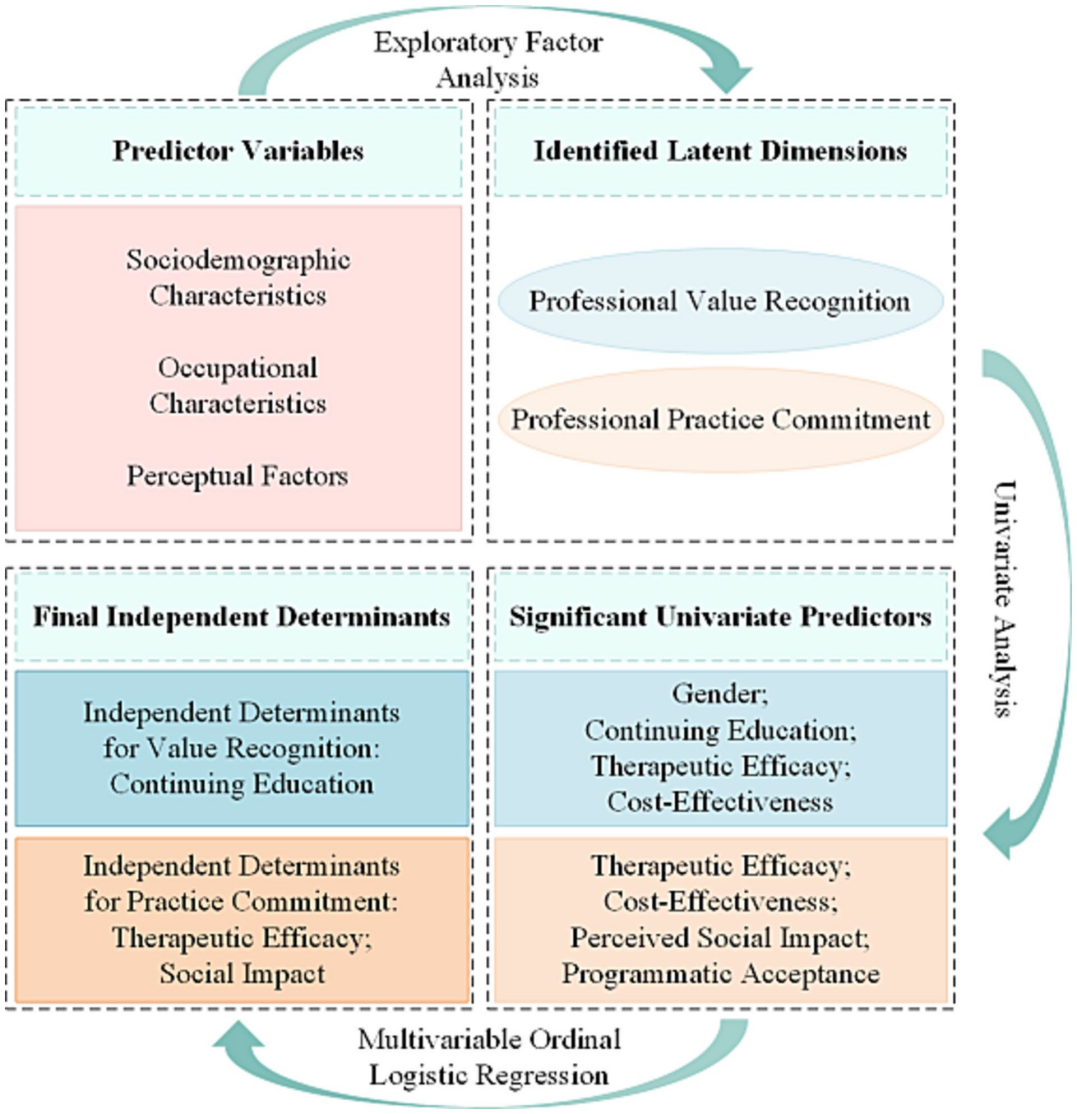
The theoretical and analytical framework of the study. The diagram outlines the analytical workflow, beginning with predictor variables, followed by the identification of latent dimensions through Exploratory Factor Analysis (EFA), and concluding with the assessment of independent predictors for each dimension via univariate and multivariable ordinal logistic regression.

Data were independently double-entered into EpiData 3.1 by two trained researchers and cross-verified to ensure accuracy. All statistical analyses were conducted using SPSS version 22.0 and Stata version 17.0. No missing values were present in the analysis variables; thus, no imputation was required. The overall analytical approach is summarized in Figure 4.

Descriptive statistics were used to summarize participants’ demographic and occupational characteristics. Normality of continuous variables was assessed using the Shapiro–Wilk test. Normally distributed variables are reported as mean ± standard deviation (x̄ ± SD), and categorical variables as frequencies and percentages [*n* (%)].

An exploratory factor analysis (EFA) was conducted to identify the latent structure of the professional identity scale within this population. Univariate ordinal logistic regression was then used to identify potential predictors for each dimension (*p* < 0.05). Two multivariable stepwise ordinal logistic regression models were subsequently developed to identify independent determinants for each dimension. The proportional odds assumption was evaluated using the Brant test (*χ*^2^ = 26.015, *p* > 0.05). All tests were two-sided, with statistical significance defined as *α* = 0.05. Effect sizes are reported as odds ratios (OR) with 95% confidence intervals (95% CIs).

## 3 Results

A total of 240 questionnaires were administered. After excluding 19 invalid responses, 221 valid responses were retained for analysis.

### 3.1 Participant characteristics and univariate analysis of factors associated with professional identity

**Figure 5:**
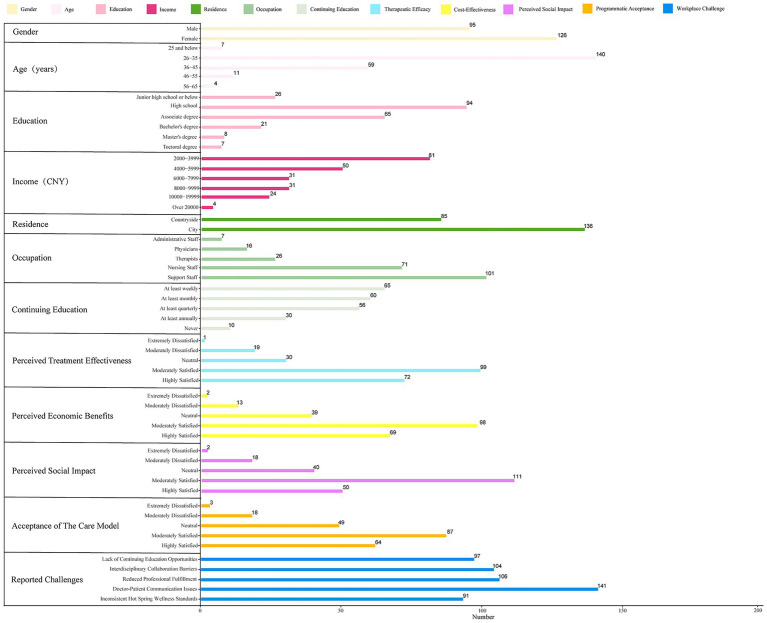
Baseline characteristics of practitioners in the HS-MLR cohort (*n* = 221).

The sociodemographic and occupational characteristics of the 221 participants are summarized in Supplementary Tables 1, 2, with a visual overview provided in Figure 5. The workforce consisted predominantly of nursing staff (32.13%) and support personnel (45.70%), together accounting for 77.83% of the sample. In contrast, clinically trained medical professionals-physicians and therapists-comprised less than 20%. The cohort was predominantly young, with 90.05% of participants between 26 and 45 years of age. Most held less than a bachelor’s degree (83.61%), and the majority were female (57.01%).

Based on the two-factor structure of professional identity identified through exploratory factor analysis, we conducted two separate univariate ordinal logistic regression analyses.

The first analysis identified several factors significantly associated with Professional Value Recognition (Table 1). These included Gender (*χ*^2^ = 10.812, *p* = 0.029), Continuing Education (*χ*^2^ = 54.321, *p* < 0.001), Therapeutic Efficacy (*χ*^2^ = 36.508, *p* = 0.002), Cost-Effectiveness (*χ*^2^ = 43.966, *p* < 0.001), and Perceived Social Impact (*χ*^2^ = 26.353, *p* = 0.049).

**Table 1:** Univariate analysis of factors associated with professional value recognition levels.

Variable	*χ*^2^ value	*p* value
Gender	10.812	**0.029** ^*^
Age	19.389	0.249
Education	30.784	0.058
Income	29.216	0.084
Residence	8.697	0.069
Occupation	8.883	0.918
Continuing Education	54.321	**<0.001** ^***^
Therapeutic Efficacy	36.508	**0.002** ^**^
Cost-Effectiveness	43.966	**<0.001** ^***^
Perceived Social Impact	26.353	**0.049** ^*^
Programmatic Acceptance	10.987	0.81
Workplace Challenges		
Lack of Continuing Education Opportunities	5.707	0.222
Interdisciplinary Collaboration Barriers	5.453	0.244
Reduced Professional Fulfillment	3.195	0.526
Doctor-Patient Communication Issues	2.176	0.703
Inconsistent Hot Spring Wellness Standards	8.728	0.068
Else	2.623	0.623

^*^p < 0.05, ^**^p < 0.01, ^***^p < 0.001. Bold values indicate statistical significance (*p* < 0.05).

The second analysis examined correlates of Professional Practice Commitment (Table 2). Therapeutic Efficacy (*χ*^2^ = 290.058, *p* < 0.001), Cost-Effectiveness (*χ*^2^ = 170.694, *p* < 0.001), Perceived Social Impact (*χ*^2^ = 196.626, *p* < 0.001), and Programmatic Acceptance (*χ*^2^ = 127.255, *p* < 0.001) all emerged as highly significant predictors.

**Table 2:** Univariate analysis of factors associated with professional practice commitment levels.

Variable	*χ*^2^ value	*p* value
Gender	9.162	0.057
Age	8.775	0.922
Education	10.752	0.952
Income	19.237	0.506
Residence	4.137	0.388
Occupation	11.206	0.797
Continuing Education	24.038	0.089
Therapeutic Efficacy	290.058	**<0.001** ^***^
Cost-Effectiveness	170.694	**<0.001** ^***^
Perceived Social Impact	196.626	**<0.001** ^***^
Programmatic Acceptance	127.255	**<0.001** ^***^
Workplace Challenges		
Lack of Continuing Education Opportunities	2.857	0.582
Interdisciplinary Collaboration Barriers	2.92	0.571
Reduced Professional Fulfillment	3.195	0.526
Doctor-Patient Communication Issues	1.644	0.801
Inconsistent Hot Spring Wellness Standards	1.494	0.828
Else	1.425	0.84

^*^p < 0.05, ^**^p < 0.01, ^***^p < 0.001. Bold values indicate statistical significance (*p* < 0.05).

### 3.2 Descriptive analysis of the two dimensions of professional identity

Based on the two-factor structure identified in our analysis, we further conducted a descriptive analysis of the two distinct dimensions of professional identity: Professional Value Recognition and Professional Practice Commitment.

For Professional Value Recognition, satisfaction levels among the 221 participants are summarized in Supplementary Table 3. Overall, participants reported moderate value recognition, with “Neutral” being the most common response (*n* = 61, 27.60%). Further breakdowns across its four sub-dimensions—Occupational Calling, Career Advancement Prospects, Work Engagement, and Occupational Prestige—are provided in Supplementary Table 4. Among these, Occupational Calling received the highest mean score (Mean = 3.10, SD = 1.245).

A similar pattern was observed for Professional Practice Commitment (Supplementary Table 5), where the “Neutral” and “Moderately Satisfied” represented the largest response categories. Descriptive statistics for its three sub-dimensions—Professional Competency Valuation, Service-Oriented Mindset, and Continuing Education Intention—are presented in Supplementary Table 6. Notably, Service-Oriented Mindset received the highest mean score across all identity dimensions (Mean = 3.71, SD = 1.068), indicating a strong service orientation among practitioners.

### 3.3 Determinants of professional identity dimensions

To identify independent determinants of each professional identity dimension, significant predictors from univariate analyses were entered into two separate multivariable stepwise ordinal logistic regression models. Both final models satisfied the proportional odds assumption, as indicated by Brant tests (Omnibus: *χ*^2^ = 26.015, *p* = 0.353).

The final model for Professional Value Recognition (Table 3) revealed that, after covariate adjusting, continuing education frequency remained the only independent predictor. Compared to those who never participated, practitioners attending training at least monthly had nearly six-fold higher odds of reporting stronger professional value recognition (OR = 5.81), while those participating at least weekly showed even greater odds (OR = 7.61).

**Table 3:** Multivariable ordinal logistic regression analysis of factors associated with professional value recognition.

Variable	*β*	OR	Standard Error	Wald *χ*^2^ value	*p* value	95% Confidence interval	
						Upper limit	Lower limit
Continuing education (ref: never)							
At least weekly	2.03	7.61	0.658	12.573	**<0.001** ^***^	1.043	3.622
At least monthly	1.76	5.81	0.646	7.134	**0.008** ^**^	0.46	2.994

^**^p < 0.01, ^***^p < 0.001. Bold values indicate statistical significance (*p* < 0.05).

For Professional Practice Commitment (Table 4), perceived treatment effectiveness, perceived social impact, and programmatic acceptance emerged as significant independent determinants. Notably, perception social impact was a particularly strong predictor: practitioners who were ‘Moderately Dissatisfied’ with social impact had 93% lower odds of expressing strong professional practice commitment compared to those who were ‘Highly Satisfied’ (OR = 0.07).

**Table 4:** Multivariable ordinal logistic regression analysis of factors associated with professional practice commitment.

Influence factors	*β*	OR	Standard Error	Wald *χ*^2^ value	*p* value	95% Confidence interval	
						Upper limit	Lower limit
Perceived treatment effectiveness (ref: highly satisfied)							
Neutral	−1.634	0.2	0.602	7.369	**0.007** ^**^	−2.814	−0.454
Perceived social impact (ref: highly satisfied)							
Moderately Dissatisfied	−2.672	0.07	0.789	11.459	**0.001** ^**^	−4.22	−1.125
Moderately Satisfied	−0.741	0.48	0.362	4.19	**0.041** ^*^	−1.45	−0.031
Programmatic acceptance (ref: highly satisfied)							
Moderately Satisfied	−0.667	0.51	0.335	3.961	**0.047** ^*^	−1.324	−0.01

^*^p < 0.05, ^**^p < 0.01. Bold values indicate statistical significance (*p* < 0.05).

**Figure 6:**
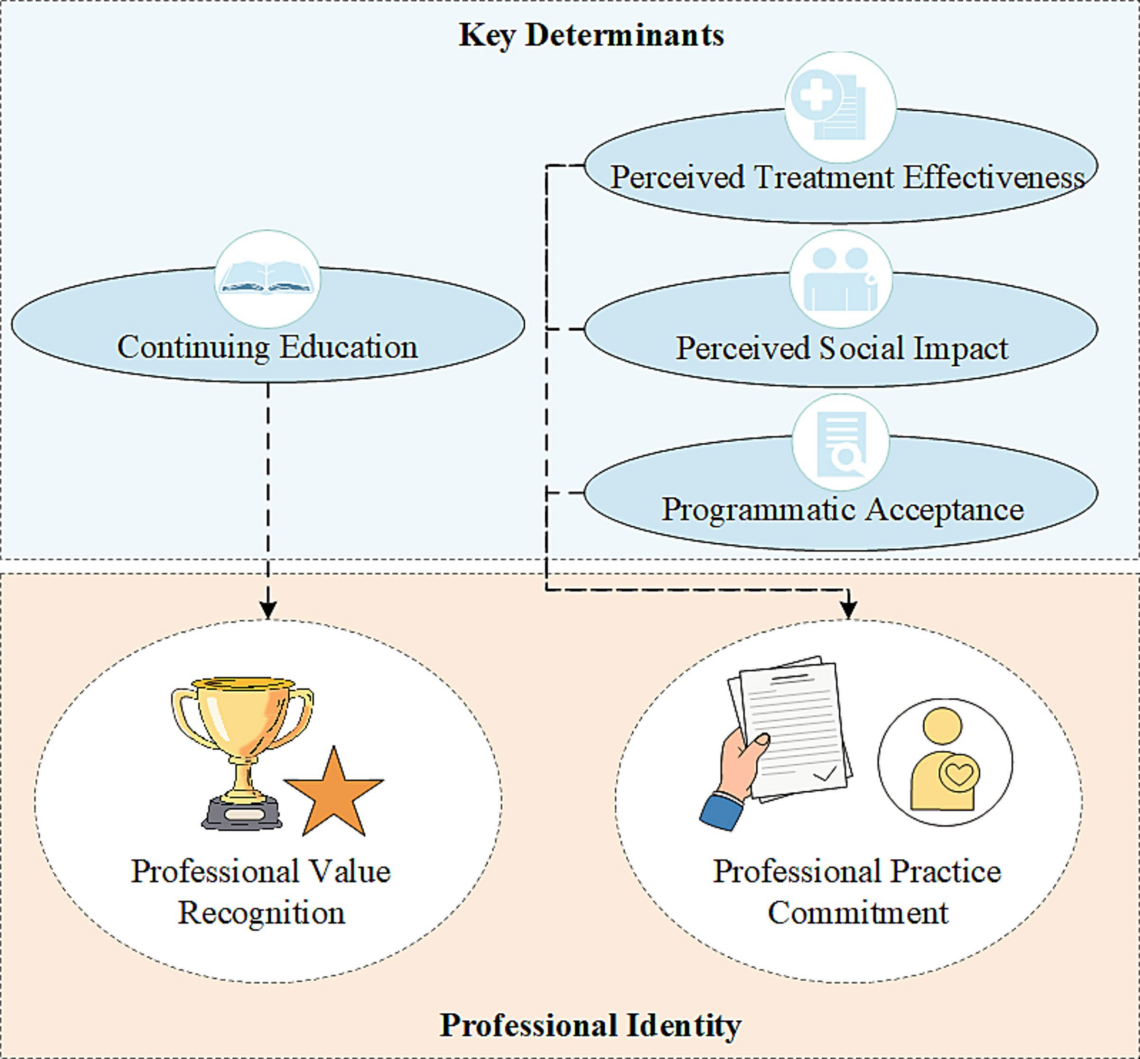
Conceptual model of the determinants of professional identity. The diagram illustrates the dual-pathway mechanism of professional identity formation identified in this study. The top panel (Key Determinants) shows that Continuing Education was the sole significant predictor for Professional Value Recognition. Concurrently, Perceived Treatment Effectiveness, Perceived Social Impact, and Programmatic Acceptance were all significant predictors for Professional Practice Commitment. The bottom panel visually represents the two distinct dimensions of Professional Identity.

To visually summarize the final dual-pathway mechanism identified from the multivariable regression analyses, a conceptual model is presented in Figure 6. This model illustrates the distinct sets of determinants that predict Professional Value Recognition and Professional Practice Commitment, respectively.

## 4 Discussion

### 4.1 Principal findings and theoretical implications

This study offers empirical evidence on the determinants of professional identity among practitioners within China’s emerging Hot Spring Medical and Long-term Care Integration with Rehabilitation Services (HS-MLR) model. A key finding is that professional identity in this group comprises two distinct dimensions: Professional Value Recognition and Professional Practice Commitment. Multivariable analyses revealed that each dimension is influenced by different factors, supporting a dual-pathway mechanism of identity formation.

Professional Value Recognition—reflecting one’s sense of vocation, prestige, and career prospects—was predicted solely by participation in continuing education. This aligns with the Professional Identity Formation (PIF) framework, which highlights ongoing development as essential to identity building (32, 40). Continuous learning not fosters knowledge acquisition but also integrates practitioners into a community of practice, facilitating the construction of professional meaning and identity (41, 42). Although economic benefits were significant predictors in univariate analyses, they did not emerge in the final model. This contrasts with conventional traditional healthcare settings, where financial incentives often underpin job satisfaction (43, 44). Our results suggest that within the experiential and service-based context of HS-MLR, intrinsic rewards from professional growth may surpass extrinsic motivations.

In contrast, Professional Practice Commitment—which encompasses professional competency and service orientation—was influenced by perceived treatment effectiveness, perceived social impact, and acceptance of the HS-MLR model. These factors align with self-efficacy and social identity theories. Confidence in treatment effectiveness reflects trust in one’s clinical skills, consistent with the work of Klassen et al. (45). Similarly, recognizing the social value of healthcare aligns with Kreindler et al. (46), who demonstrated that physicians’ professional identity is strongly tied to the perceived social benefits of patient recovery.

The broader applicability of this dual-pathway model is especially relevant to interdisciplinary health service settings like HS-MLR, where clinical rehabilitation converges with wellness and hospitality. In contrast to traditional unidimensional identity frameworks, our model more effectively captures the complexity of practitioners in hybrid roles. Given the heightened potential for role ambiguity in such settings (47), distinguishing between the “why” (Value Recognition) and the “how” (Practice Commitment) of professional identity provides a clearer framework for understanding identity negotiation processes and strengthening professional resilience (48).

### 4.2 Practical implications for workforce management and practitioners

The dual-pathway model offers practical implications for evidence-based human resource management (HRM) and practitioner development. For managers, it provides a diagnostic tool to shift from generalized, control-oriented HRM strategies toward more effective, commitment-based approaches (8). By distinguishing whether challenges stem from low Professional Value Recognition or weak Professional Practice Commitment, leaders can design targeted interventions. For instance, when value recognition is inadequate, effort should emphasize continuous learning and clear career advancement opportunities. Conversely, when practice commitment is low, strategies should aim to improve service efficacy and effectively communicate the social and therapeutic impact of practitioners’ work. This tailored approach overcomes the limitations of generic supervisor training, which often lacks contextual relevance and fails to produce meaningful outcomes (49).

For practitioners, the model offers a reflective framework to strengthen self-awareness and professional resilience. Recognizing the two dimensions of identity enables individuals to better pinpoint sources of occupational challenges. Notably, our finding that continuing education is the sole predictor of Professional Value Recognition suggests that active participation in lifelong learning directly enhances one’s sense of professional worth. This aligns with the concept of cognitive mastery proposed by Guraya et al. (50), positioning continuous learning as fundamental to resilience and essential for sustaining professional identity and long-term career adaptability.

### 4.3 Limitations

Although this study provides valuable insights, several methodological limitations should be noted to appropriately contextualize the findings. First, the use of a non-probability convenience sampling approach limits the generalization of the results. Notwithstanding its broad geographic coverage, a substantial proportion of participants were concentrated in provinces with developed hot spring industries, potentially introducing regional bias. Moreover the sample was predominantly drawn from public institutions (75.1%), which may not fully represent the diversity of institutional ownership within this sector. Second, the cross-sectional design precludes causal inferences. The associations identified should be interpreted as correlational rather than causal. Future studies should adopt longitudinal designs to more rigorously examine the temporal causal and dynamic pathways shaping professional identity.

### 4.4 Global relevance and future directions

Notwithstanding these limitations, this study provided valuable insights into an emerging field of global significance. Balneology and hot spring therapy are already integral to mainstream medical practice in several European countries and Japan (9). In these settings, a well-established hot spring industry delivers not only therapeutic benefits but also considerable economic value (51, 52), and is often embedded within national healthcare systems—exemplified by France, where thermal treatments are routinely used in burn rehabilitation and covered by social insurance (53). The sustainability of such models relies on a skilled and stable professional workforce, with prior research underscoring that investment in qualified personnel is crucial for long-term success (52). By identifying determinants of professional identity among practitioners in China’s hot spring sector, this study lays a critical foundation for targeted strategies to strengthen this workforce. These efforts are essential not only for advancing China’s HS-MLR model sustainably, but also for informing the development of similar integrated care systems globally. Future research should focus on designing and evaluating dual-pathway interventions that enhance professional identity, reduce burnout, and mitigate turnover, thereby supporting the resilience and sustainability of this specialized workforce.

## 5 Conclusion

This study establishes, for the first time, that professional identity within China’s Hot Spring Medical and Long-term Care Integration with Rehabilitation Services (HS-MLR) model comprises two distinct dimension—Professional Value Recognition and Professional Practice Commitment—each influenced by unique determinants. The findings that continuing education drives value recognition, while perceived efficacy and social value predict practice commitment, offer actionable insights for policy and management. These results provide an empirical basis for shaping strategies aimed at strengthening this specialized workforce. Therefore, future efforts should be directed toward applying these insights to inform the design of targeted training programs and the necessary service reorganization needed to enhance practitioners’ professional identity. By addressing these specific needs—namely, fostering professional development and restructuring services to reinforce perceived value—the long-term workforce sustainability and service quality within this emerging sector can be substantially improved.

## Data Availability

The datasets presented in this article are not readily available because the datasets generated and analyzed during the current study are not publicly available due to institutional and ethical restrictions, as they contain information that could compromise the privacy of individual participants. Data may be made available from the corresponding author upon reasonable request and with permission from the Ethics Committee of the Second Affiliated Hospital of Kunming Medical University. Requests to access the datasets should be directed to Xiaochen Li, 2691447640@qq.com.
