# Supplementary material for: Determinants of professional identity among practitioners in China’s hot spring-integrated care model
Source: Front Med (Lausanne). 2025 Dec 10;12:1650338. doi: 10.3389/fmed.2025.1650338 (PMC12730160; doi:10.3389/fmed.2025.1650338)
Supplement: Supplementary file 1 [file Data_Sheet_1.pdf]

Supplementary Table 1 Distribution of Professional Value Recognition Levels Across Participant Subgroups

Items	Number	Professional Value Recognition				
		Extremely Dissatisfied	Moderately Dissatisfied	Neutral	Moderately Satisfied	Highly Satisfied
**Gender**						
Male	95	4(4.21%)	23(24.21%)	23(24.21%)	18(18.95%)	27(28.42%)
Female	126	1(0.79%)	30(23.81%)	38(30.16%)	38(30.16%)	19(15.08%)
**Age**						
25 and below	7	0	1(14.29%)	3(42.86%)	2(28.57%)	1(14.29%)
26-35	140	4(2.86%)	39(27.86%)	44(31.43%)	26(18.57%)	27(19.29%)
36-45	59	1(1.69%)	11(18.64%)	11(18.64%)	20(33.90%)	16(27.12%)
46-55	11	0	2(18.18%)	2(18.18%)	5(45.45%)	2(18.18%)
56-65	4	0	0	1(25.00%)	3(75.00%)	0
**Education**						
Junior high school or below	26	0	2(7.69%)	5(19.23%)	8(30.77%)	11(42.31%)
High school	94	1(1.06%)	22(23.40%)	32(34.04%)	23(24.47%)	16(17.02%)
Associate degree	65	3(4.62%)	22(33.85%)	19(29.23%)	12(18.46%)	9(13.85%)
Bachelor's degree	21	1(4.77%)	6(28.57%)	2(9.52%)	7(33.33%)	5(23.81%)
Master's degree	8	0	1(12.50%)	2(25.00%)	2(25.00%)	3(37.50%)
Toctoral degree	7	0	0	1(14.29%)	4(57.14%)	2(28.57%)
**Income**						
2000-3999	81	0	18(22.22%)	21(25.93%)	24(29.63%)	18(22.22%)
4000-5999	50	2(4.00%)	8(16.00%)	17(34.00%)	12(24.00%)	11(22.00%)
6000-7999	31	0	13(41.94%)	9(29.03%)	4(12.90%)	5(16.13%)
8000-9999	31	0	7(22.58%)	7(22.58%)	10(32.26%)	7(22.58%)
10000-19999	24	3(12.50%)	6(25.00%)	7(29.17%)	5(20.83%)	3(12.50%)
Over 20000	4	0	1(25.00%)	0	1(25.00%)	2(50.00%)
**Residence**						
Countryside	136	3(2.21%)	26(19.12%)	34(25.00%)	40(29.41%)	33(24.26%)
City	85	2(2.35%)	27(31.76%)	27(31.76%)	16(18.82%)	13(15.29%)
**Occupation**						
Administrative Staff	7	0	2(28.57%)	2(28.57%)	2(28.57%)	1(14.29%)
Physicians	16	0	4(25.00%)	2(12.50%)	4(25.00%)	6(37.50%)
Therapists	26	1(3.85%)	7(26.92%)	7(26.92%)	8(30.77%)	3(11.54%)
Nursing Staff	71	2(2.82%)	17(23.94%)	17(23.94%)	21(29.58%)	14(19.72%)
Support Staff	101	2(2.00%)	23(22.77%)	33(32.67%)	21(20.79%)	22(21.78%)
**Continuing Education**						
At least weekly	65	0	7(10.77%)	10(15.38%)	29(44.62%)	19(29.23%)
At least monthly	60	0	17(28.33%)	11(18.33%)	14(23.33%)	18(30.00%)
At least quarterly	56	2	17(30.36%)	22(39.28%)	8(14.29%)	7(12.50%)
At least annually	30	2(6.67%)	9(30.00%)	13(43.33%)	4(13.33%)	2(6.67%)
Never	10	1(10.00%)	3(30.00%)	5(50.00%)	1(10.00%)	0
**Therapeutic Efficacy**						
Extremely Dissatisfied	1	0	0	0	0	1(100.00%)

Moderately Dissatisfied	19	0	5(26.32%)	9(47.37%)	4(21.05%)	1(5.26%)
Neutral	30	1(3.33%)	8(26.67%)	12(40.00%)	5(16.67%)	4(13.33%)
Moderately Satisfied	99	1(1.01%)	29(29.29%)	32(32.32%)	21(21.21%)	16(16.16%)
Highly Satisfied	72	3(4.17%)	11(15.28%)	8(11.11%)	26(36.11%)	24(33.33%)
**Cost-Effectiveness**						
Extremely Dissatisfied	2	0	1(50.00%)	0	0	1(50.00%)
Moderately Dissatisfied	13	0	6(46.15%)	4(30.77%)	3(23.08%)	0
Neutral	39	1(2.56%)	7(17.95%)	22(56.41%)	7(17.95%)	2(5.13%)
Moderately Satisfied	98	1(1.02%)	29(29.59%)	25(25.51%)	20(20.41%)	23(23.47%)
Highly Satisfied	69	3(4.35%)	10(14.49%)	10(14.49%)	26(37.68%)	20(28.99%)
**Perceived Social Impact**						
Extremely Dissatisfied	2	0	0	0	1(50.00%)	1(50.00%)
Moderately Dissatisfied	18	0	6(33.33%)	9(50.00%)	2(11.11%)	1(5.56%)
Neutral	40	1(2.50%)	11(27.50%)	14(35.00%)	10(25.00%)	4(10.00%)
Moderately Satisfied	111	2(1.80%)	26(23.42%)	33(29.73%)	28(25.23%)	22(19.82%)
Highly Satisfied	50	2(4.00%)	10(20.00%)	5(10.00%)	15(30.00%)	18(36.00%)
**Programmatic Acceptance**						
Extremely Dissatisfied	3	0	1(33.33%)	1(33.33%)	0	1(33.33%)
Moderately Dissatisfied	18	0	5(27.78%)	6(33.33%)	4(22.22%)	3(16.67%)
Neutral	49	2(4.08%)	10(20.41%)	18(36.73%)	12(24.48%)	7(14.29%)
Moderately Satisfied	87	1(1.15%)	22(25.29%)	22(25.29%)	26(29.89%)	16(18.39%)
Highly Satisfied	64	2(3.13%)	15(23.44%)	14(21.88%)	14(21.88%)	19(29.69%)
**Workplace Challenges**						
Lack of Continuing Education Opportunities	97	4(4.12%)	23(23.71%)	28(28.87%)	19(19.59%)	23(23.71%)
Interdisciplinary Collaboration Barriers	104	4(3.85%)	28(26.92%)	25(24.04%)	29(27.88%)	18(17.31%)
Reduced Professional Fulfillment	106	2(1.89%)	27(25.47%)	34(32.08%)	24(22.64%)	19(18.27%)
Doctor-Patient Communication Issues	141	2(1.42%)	36(25.53%)	37(26.24%)	35(24.82%)	31(21.99%)
Inconsistent Hot Spring Wellness Standards	91	2(2.20%)	15(16.48%)	22(24.18%)	27(29.67%)	25(27.47%)
Else	10	0	1(10.00%)	2(20.00%)	4(40.00%)	3(30.00%)

Supplementary Table 2 Distribution of Professional Value Recognition Levels Across Participant Subgroups

Items	Number	Professional Practice Commitment				
		Extremely Dissatisfied	Moderately Dissatisfied	Neutral	Moderately Satisfied	Highly Satisfied
**Gender**						
Male	95	1 (1.05%)	7 (7.37%)	20 (21.05%)	27 (28.42%)	40 (42.11%)
Female	126	0	12 (9.52%)	26 (20.63%)	55 (43.65%)	33 (26.19%)
**Age**						

25 and below	7	0	2 (28.57%)	0	2 (28.57%)	3(42.86%)
26-35	140	1 (0.71%)	10 (7.14%)	31 (22.14%)	50 (35.71%)	48 (34.29%)
36-45	59	0	5	11	25	18
46-55	11	0	1	3	4	3
56-65	4	0	1	1	1	1
**Education**						
Junior high school or below	26	0	4(15.38%)	5(19.23%)	9(34.62%)	8(30.77%)
High school	94	1 (1.06%)	8 (8.51%)	21 (22.34%)	31 (32.98%)	33 (35.11%)
Associate degree	65	0	6 (9.23%)	14 (21.54%)	24 (36.92%)	21 (32.31%)
Bachelor's degree	21	0	1 (4.76%)	4 (19.05%)	8 (38.10%)	8 (38.10%)
Master's degree	8	0	0	1 (12.50%)	6 (75.00%)	1 (12.50%)
Toctoral degree	7	0	0	1 (14.29%)	4 (57.14%)	2 (28.57%)
**Income**						
2000-3999	81	0	7 (8.64%)	16 (19.75%)	35 (43.21%)	23 (28.40%)
4000-5999	50	0	8 (16.00%)	12 (24.00%)	12 (24.00%)	18 (36.00%)
6000-7999	31	1 (3.23%)	3 (9.68%)	7 (22.58%)	9 (29.03%)	11 (35.48%)
8000-9999	31	0	0	5 (16.13%)	14 (45.16%)	12 (38.71%)
10000-19999	24	0	1 (4.17%)	5 (20.83%)	10 (41.67%)	8 (33.33%)
Over 20000	4	0	0	1 (25.00%)	2 (50.00%)	1 (25.00%)
**Residence**						
Countryside	136	0	10 (7.35%)	25 (18.38%)	54 (39.71%)	47 (34.56%)
City	85	1 (1.18%)	9 (10.59%)	21 (24.71%)	28 (32.94%)	26 (30.59%)
**Occupation**						
Administrative Staff	7	0	0	3 (42.86%)	1 (14.29%)	3 (42.86%)
Physicians	16	0	0	2 (12.50%)	7 (43.75%)	7 (43.75%)
Therapists	26	0	1 (3.85%)	6 (23.08%)	12 (46.15%)	7 (26.92%)
Nursing Staff	71	1 (1.41%)	8 (11.27%)	16 (22.54%)	24 (33.80%)	22 (30.99%)
Support Staff	101	0	10 (9.90%)	19 (18.81%)	38 (37.62%)	34 (33.66%)
**Continuing Education**						
At least weekly	65	0	10 (15.38%)	9(13.85%)	28(43.08%)	18(27.69%)
At least monthly	60	0	2(3.33%)	12(20.00%)	23(38.33%)	23(38.33%)
At least quarterly	56	0	4(7.14%)	13(23.21%)	23(41.07%)	16(28.57%)
At least annually	30	1	2(6.67%)	10(33.33%)	7(23.33%)	10(33.33%)
Never	10	0	1(10.00%)	2(20.00%)	1(10.00%)	6(60.00%)
**Therapeutic Efficacy**						
Extremely Dissatisfied	1	1 (100.00%)	0	0	0	0
Moderately Dissatisfied	19	0	6 (31.58%)	8 (42.11%)	5 (26.32%)	0
Neutral	30	0	7 (23.33%)	14 (46.67%)	5 (16.67%)	4 (13.33%)
Moderately Satisfied	99	0	6 (6.06%)	17 (17.17%)	45 (45.45%)	31 (31.31%)
Highly Satisfied	72	0	0	7 (9.72%)	27 (37.50%)	38 (52.78%)
**Cost-Effectiveness**						
Extremely Dissatisfied	2	1 (50.00%)	0	1 (50.00%)	0	0
Moderately Dissatisfied	13	0	4 (30.77%)	5 (38.46%)	4 (30.77%)	0
Neutral	39	0	10 (25.64%)	14 (35.90%)	11 (28.21%)	4 (10.26%)

Moderately Satisfied	98	0	5 (5.10%)	21 (21.43%)	38 (38.78%)	34 (34.69%)
Highly Satisfied	69	0	0	5 (7.25%)	29 (42.03%)	35 (50.72%)
**Perceived Social Impact**						
Extremely Dissatisfied	2	1 (50.00%)	0	1 (50.00%)	0	0
Moderately Dissatisfied	18	0	9 (50.00%)	6 (33.33%)	3 (16.67%)	0
Neutral	40	0	7 (17.50%)	15 (37.50%)	11 (27.50%)	7 (17.50%)
Moderately Satisfied	111	0	3 (2.70%)	22 (19.82%)	49 (44.14%)	37 (33.33%)
Highly Satisfied	50	0	0	2 (4.00%)	19 (38.00%)	29 (58.00%)
**Programmatic Acceptance**						
Extremely Dissatisfied	3	1 (33.33%)	0	2 (66.67%)	0	0
Moderately Dissatisfied	18	0	7 (38.89%)	6 (33.33%)	5 (27.78%)	0
Neutral	49	0	6 (12.24%)	14 (28.57%)	19 (38.78%)	10 (20.41%)
Moderately Satisfied	87	0	5 (5.75%)	17 (19.54%)	37 (42.53%)	28 (32.18%)
Highly Satisfied	64	0	1 (1.56%)	7 (10.94%)	21 (32.81%)	35 (54.69%)
**Workplace Challenges**						
Lack of Continuing Education Opportunities	97	1 (1.03%)	9 (9.28%)	23 (23.71%)	32 (32.99%)	32 (32.99%)
Interdisciplinary Collaboration Barriers	104	1 (0.96%)	8 (7.69%)	21 (20.19%)	43 (41.35%)	31 (29.81%)
Reduced Professional Fulfillment	106	0	12 (11.32%)	23 (21.70%)	37 (34.91%)	34 (32.08%)
Doctor-Patient Communication Issues	141	1 (0.71%)	11 (7.80%)	27 (19.15%)	54 (38.30%)	48 (34.04%)
Inconsistent Hot Spring Wellness Standards	91	0	7 (7.69%)	19 (20.88%)	32 (35.16%)	33 (36.26%)
Else	10	0	0	3 (30.00%)	4 (40.00%)	3 (30.00%)

Supplementary Table 3 Distribution of staff professional value recognition levels.

Professional Value Recognition	Score Range (points)	n (Cases)	Proportion (%)
Extremely Dissatisfied	1-4	5	2.26
Moderately Dissatisfied	5-8	53	23.98
Neutral	9-12	61	27.60
Moderately Satisfied	13-16	56	25.34
Highly Satisfied	17-20	46	20.81

Supplementary Table 4 Scores and rankings of staff professional value recognition across dimensions

Dimension	Score			
	Range	Means	SD	Rank
Occupational Calling	1-5	3.1	1.245	1
Career Advancement Prospects	1-5	3.05	1.303	3
Work Engagement	1-5	2.98	1.259	4
Occupational Prestige	1-5	3.09	1.229	2

Supplementary Table 5 Distribution of staff professional practice commitment levels.

Professional Practice Commitment	Score Range (points)	n (Cases)	Proportion (%)
Extremely Dissatisfied	1-3	1	0.45
Moderately Dissatisfied	4-6	19	8.60
Neutral	7-9	46	20.81
Moderately Satisfied	10-12	82	37.10
Highly Satisfied	13-15	73	33.03

Supplementary Table 6 Scores and rankings of staff professional practice commitment across dimensions

Dimension	Score			
	Range	Means	SD	Rank
Professional Competency Valuation	1-5	3.6	1.212	3
Service-Oriented Mindset	1-5	3.71	1.068	1
Continuing Education Intention	1-5	3.67	1.077	2
