# Supplementary material for: Determinants of professional identity among practitioners in China’s hot spring-integrated care model
Source: Front Med (Lausanne). 2025 Dec 10;12:1650338. doi: 10.3389/fmed.2025.1650338 (PMC12730160; doi:10.3389/fmed.2025.1650338)
Supplement: Supplementary file 2 [file Data_Sheet_2.pdf]

Survey on Practitioners in Chinese Hot Spring Medical and Long-term Care Institutions

Dear Respondent,
Greetings. We are a research team dedicated to exploring and understanding the operational landscape and challenges within China's Hot Spring Medical and Long-term Care Integration with Rehabilitation Services (HS-MLR) model. As society increasingly pursues healthier lifestyles, this integrated model, which combines leisure, wellness, and rehabilitation, is becoming an ideal choice for urban populations seeking physical and mental relaxation and recovery.

To more deeply understand the attitudes and expectations of practitioners towards the HS-MLR model and to identify potential operational barriers, we cordially invite you to participate in this survey. Your valuable input will help us formulate targeted strategies to enhance workforce sustainability, providing crucial references for the industry. This will also contribute to service optimization and meeting the personalized needs of consumers.

Please be assured that this survey is anonymous. All collected data will be used exclusively for academic research and market analysis. We will strictly adhere to data protection regulations to ensure the security of your personal information.

This survey will take approximately 5-10 minutes to complete. We sincerely appreciate you taking the time to contribute to this study. Your responses are invaluable to the development of the hot spring-integrated care sector. Should you have any questions during the process, please do not hesitate to contact our research team.

Let us work together to advance the hot spring-integrated care industry towards a more professional and health-oriented future, bringing high-quality wellness experiences to more consumers.

Thank you once again for your support and cooperation.

1. What is your gender? ( )
   A. Male
   B. Female

2. What is your age group? ( )
   A. 25 and below
   B. 26-35
   C. 36-45

D. 46-55
E. 56-65
F. Over 65

3. What is your highest level of education? ( )
A. Junior high school or below
B. High school
C. Associate degree
D. Bachelor's degree
E. Master's degree
F. Doctoral degree

4. What is your monthly income level (in CNY)? ( )
A. Below 2000
B. 2000-3999
C. 4000-5999
D. 6000-7999
E. 8000-9999
F. 10000-19999
G. Over 20000

5. What is your residential area? ( )
A. City
B. Countryside

6. What is your occupation? ( )
A. Administrative Staff
B. Physicians
C. Therapists
D. Nursing Staff
E. Support Staff
F. Other, please specify: ________________

7. How often does your institution provide you with continuing education? ( )
A. At least weekly
B. At least monthly
C. At least quarterly
D. At least annually
E. Never

8. Please indicate your level of agreement with the following statement: "I believe that hot spring therapy itself has unique, irreplaceable therapeutic effects that are highly beneficial to health." ( )
A. Strongly disagree

B. Disagree
C. Neutral
D. Agree
E. Strongly agree

9. Please indicate your level of agreement with the following statement: "I believe that hot spring-integrated care can more fully leverage the therapeutic effects of hot springs to achieve a more targeted approach to resolving health problems." ( )
A. Strongly disagree
B. Disagree
C. Neutral
D. Agree
E. Strongly agree

Section: Perceptions of Hot Spring + Western Medicine Rehabilitation Projects

10. How would you rate your level of approval for "Hot Spring + Western Medicine Rehabilitation" projects? ( )
A. Very low B. Low C. Moderate D. High E. Very high

11. In your opinion, what is the level of contribution of "Hot Spring + Western Medicine Rehabilitation" projects to the institution's public reputation? ( )
A. Very low B. Low C. Moderate D. High E. Very high

12. In your opinion, what is the level of contribution of "Hot Spring + Western Medicine Rehabilitation" projects to the institution's profitability? ( )
A. Very low B. Low C. Moderate D. High E. Very high

Section: Perceptions of Hot Spring + Traditional Chinese Medicine (TCM) Rehabilitation Projects

13. How would you rate your level of approval for "Hot Spring + TCM Rehabilitation" projects? ( )
A. Very low B. Low C. Moderate D. High E. Very high

14. In your opinion, what is the level of contribution of "Hot Spring + TCM Rehabilitation" projects to the institution's public reputation? ( )
A. Very low B. Low C. Moderate D. High E. Very high

15. In your opinion, what is the level of contribution of "Hot Spring + TCM Rehabilitation" projects to the institution's profitability? ( )
A. Very low B. Low C. Moderate D. High E. Very high

Section: Perceptions of Hot Spring + Non-medical Intervention Rehabilitation

Projects

16. How would you rate your level of approval for "Hot Spring + Non-medical Intervention" projects? ( )
A. Very low B. Low C. Moderate D. High E. Very high

17. In your opinion, what is the level of contribution of "Hot Spring + Non-medical Intervention" projects to the institution's public reputation? ( )
A. Very low B. Low C. Moderate D. High E. Very high

18. In your opinion, what is the level of contribution of "Hot Spring + Non-medical Intervention" projects to the institution's profitability? ( )
A. Very low B. Low C. Moderate D. High E. Very high

Section: Work Attitudes

19. Please indicate your level of agreement with the statement: "I believe my work is noble and helps others." ( )
A. Strongly disagree B. Disagree C. Neutral D. Agree E. Strongly agree

20. Please indicate your level of agreement with the statement: "I believe my job has promising career prospects." ( )
A. Strongly disagree B. Disagree C. Neutral D. Agree E. Strongly agree

21. Please indicate your level of agreement with the statement: "I always maintain a positive attitude in my work." ( )
A. Strongly disagree B. Disagree C. Neutral D. Agree E. Strongly agree

22. Please indicate your level of agreement with the statement: "My work is always respected by others." ( )
A. Strongly disagree B. Disagree C. Neutral D. Agree E. Strongly agree

23. Please indicate your level of agreement with the statement: "I believe that professional competence is crucial in my line of work." ( )
A. Strongly disagree B. Disagree C. Neutral D. Agree E. Strongly agree

24. Please indicate your level of agreement with the statement: "If given the opportunity in the future, I would like to pursue further study to enhance my education and skills." ( )

A. Strongly disagree B. Disagree C. Neutral D. Agree E. Strongly agree

Section: Workplace Challenges

25. What do you perceive to be the main challenges in your work? (Multiple selections are permitted) ( )

A. Lack of sufficient training and continuing education opportunities

B. Difficulties in interdisciplinary collaboration, leading to high communication costs among team members

C. Low number of consumers, resulting in a diminished sense of professional achievement

D. Excessively high consumer expectations, leading to difficulties in practitioner-patient communication

E. Inconsistent standards for hot spring-integrated care, with a lack of guidelines for symptomatic treatment

F. Other, please specify: ________________
